# Landauer Principle and Einstein Synchronization of Clocks: Ramsey Approach

**DOI:** 10.3390/e27070697

**Published:** 2025-06-29

**Authors:** Edward Bormashenko, Michael Nosonovsky

**Affiliations:** 1 Department of Chemical Engineering, Biotechnology and Materials, Engineering Sciences Faculty, Ariel University, Ariel 407000, Israel; 2 Department of Mechanical Engineering, University of Wisconsin-Milwaukee, Milwaukee, WI 53211, USA; nosonovs@uwm.edu

**Keywords:** Landauer bound, synchronization of clocks, Einstein synchronization, Ramsey theory, lattice of clocks, complete graph, transitivity

## Abstract

We introduce a synchronization procedure for clocks based on the Einstein–Landauer framework. Clocks are modeled as discrete, macroscopic devices operating at a thermal equilibrium temperature *T*. Synchronization is achieved by transmitting photons from one clock to another; the absorption of a photon by a clock reduces the uncertainty in its timekeeping. The minimum energy required for this reduction in uncertainty is determined by the Landauer bound. We distinguish between the time-bearing and non-time-bearing degrees of freedom of the clocks. A reduction in uncertainty under synchronization in the time-bearing degrees of freedom necessarily leads to heat dissipation in the non-time-bearing ones. The minimum energy dissipation in these non-time-bearing degrees of freedom is likewise given by the Landauer limit. The same is true for mechanical synchronization of clocks. We also consider lattices of clocks and analyze synchronization using a Ramsey graph approach. Notably, clocks operating at the same temperature may be synchronized using photons of different frequencies. Each clock is categorized as either synchronized or non-synchronized, resulting in a bi-colored complete graph of clocks. By Ramsey’s theorem, such a graph inevitably contains a triad (or loop) of clocks that are either all synchronized or all non-synchronized. The extension of the Ramsey approach to infinite lattices of clocks is reported.

## 1. Introduction

The synchronization of clocks is considered one of the most fundamental problems in physics because it lies at the very foundation of how we understand time, simultaneity, and ultimately the structure of space-time itself [1,2]. Clock synchronization underpins our entire framework for defining when and where events occur in physics [1,2]. Various procedures enabling the synchronization of clocks were suggested. Antiphase synchronization was the phenomenon observed by Huygens in XVII century [3,4]. Generally speaking, in Newtonian–Lagrangian mechanics, the synchronization of clocks is a trivial, straightforward procedure, because time is considered absolute, meaning it flows the same for all observers, regardless of their state of motion or location. In classical mechanics, time is considered absolute, homogeneous, and universal [5,6,7]. Clocks at different locations are assumed to tick at the same rate and show the same time, provided they were set/synchronized identically [5,6,7].

The situation is quite different in special and general relativity [8,9,10,11,12]. Synchronized clocks could not be moved from one point to another without intervention into their operation. Two solutions were suggested for synchronization of the clocks in special relativity: (i) Einstein lattice of synchronized clocks [1,2,3,4] and (ii) Eddington slow clock transport [8,9]. We followed the more comprehensive Einstein synchronization procedure. Einstein synchronization implies the following steps: (i) Assume there are two clocks, clock “A” and clock “B”, located at different positions in space but at rest relative to each other in an inertial frame. At time $\tau_{A}$ on clock labeled “A”, a light signal is sent from clock “A” to the clock “B.” (ii) At the next stage we receive the signal: the light signal reaches clock “B” at time $\tau_{B}$ (registered according to the clock “B” time). (iii) At the next stage the signal is reflected back from clock “B” to clock “A”. (iv) The reflected signal is received back at the clock A at time $\tau_{A}^{\prime }$ (fixed according to clock “A”).

Assuming the speed of light is constant and the same in both directions, the time it takes for light to travel from “A” to “B” is equal to the time it takes to return from B to A. So, $\tau_{B}=\frac{\tau_{A}+\tau_{A}^{\prime }}{2}$. If this equation is true, this means that clock “B” is synchronized with clock “A”. Assumptions behind the procedure are summarized as follows: (a) Light travels at constant speed *c* in a vacuum in all directions, (b) clocks are stationary in the same inertial frame, and (c) the time taken for the light to go from clock “A” to “B” is the same as from clock “B” to “A”. However, implicitly, one more assumption is made, namely we adopted that any light signal sent from one clock to another is suitable for the synchronization, whatever its wavelength (frequency). We demonstrated that this assumption contradicts the Landauer Principle.

The Landauer Principle is one of the limiting physical principles, which constraints the behavior of computing systems. The Landauer Principle restricts the minimal energy necessary for erasure of one bit of information. Rolf Landauer adopted that computation is a physical process; thus, it must obey the laws of physics and, first and foremost, the laws of thermodynamics [13,14,15,16,17]. This thinking led to the new limiting physical principle, establishing minimal energy cost for erasure of a single memory bit for the system operating at the equilibrium temperature *T*. The minimum amount of heat/energy W dissipated when erasing one bit of information is given by Equation (1):

(1) $$W=k_{B}Tln2,$$

where $k_{B}$ is the Boltzmann constant. The Landauer Principle also led to the fundamentally important distinction between the logic and thermodynamic irreversibility [13,14,15,16,17,18,19,20,21,22,23,24,25,26,27,28,29,30,31]. It should be emphasized that the Landauer bound, given by Equation (1), is related only to a single information-bearing degree of freedom of the entire computing system. The Landauer Principle was rigorously microscopically derived without direct reference to the Second Law of Thermodynamics [18]. A quantum mechanics extension of the Landauer Principle was demonstrated [19]. Also a relativistic generalization of the Landauer Principle was introduced [22,24]. An extension of the Landauer Principle to the many-valued logic-based computation was reported [23]. We introduce the analogy between the computers and physical clocks and extend the Landauer Principle to the Einstein synchronization of clocks.

We will also address the lattices of clocks, which are seen within the perspective of the Ramsey theory [32,33,34,35,36,37,38,39]. Ramsey theory is a branch of discrete mathematics within combinatorics that deals with the emergence of order within seemingly chaotic or random structures, provided those structures are sufficiently large. It addresses questions of the form: “How large must a structure be to guarantee that a specific property or pattern inevitably appears within it?” [32,33,34,35,36,37,38,39].

## 2. Results

### 2.1. Synchronization of Clocks Operating at the Same Temperature

Consider a pair of clocks to be synchronized, the clocks are numbered “1” and “2” correspondingly. The clocks are seen as “synchronized” or, alternatively, “non-synchronized”. Now we make the main assumptions of the suggested approach: (i) the clocks are in the thermal equilibrium with surroundings, and the equilibrium temperature of both clocks is *T*. (ii) The minimal energy necessary for the clocks’ synchronization is established with the Landauer bound, namely $E_{synchr}=k_{B}Tln2$.

We follow the general Landauer approach [13,14,15,16,17,18,19,20]. The clock is the physical device. It contains (1) the timekeeping element (oscillator), which provides a regular, consistent time interval (the “heartbeat” of the clock), such as a pendulum, balance wheel and spring, quartz crystal (in quartz clocks and watches), atomic oscillator (in atomic clocks); (2) a power source, which drives the timekeeping element and other mechanisms; (3) an escapement mechanism, which controls and regulates the release of power to the timekeeping element, converting continuous energy into discrete impulses, which translates the motion of the oscillator into usable time intervals (seconds, minutes, hours, etc.); and (4) a display or indication mechanism.

What is common between computer and clocks? Both of them are *discrete physical devices*. Both systems move through a sequence of states. The measurement of time is established by comparing their timekeeping against a known standard or reference, which may be International Atomic Time (TAI), based on the vibrations of cesium atoms or Coordinated Universal Time (UTC). Whatever is the presumed time standard, the time measurement is necessarily a discrete procedure.

A computer, in turn, moves through discrete computational states as it executes each instruction. Both of them have essential and supplementary degrees of freedom. In computers, we distinguish between the “information-bearing” and “non-information bearing” degrees of freedom [16]. Information-bearing degrees of freedom are specific physical states used to encode information in a computer, i.e., the charge of a capacitor (in DRAM, a memory chip that depends upon an applied voltage to keep the stored data), magnetization of a domain (in hard drives), and high/low voltage (in logic gates). These degrees of freedom can carry logical bits: “0” or “1”. Non-information-bearing degrees of freedom (e.g., atomic positions subject to thermal vibrations) do not directly represent logical information, i.e., atomic motions and electron energy distributions unrelated to logic states. These degrees of freedom can still absorb or carry energy but not logical bits. When you erase a bit (e.g., reset a memory location to “0” regardless of previous value), you reduce the number of possible logical states from two (“0” or “1”) to one (just “0”). This reduction in logical entropy must be compensated by an increase in physical entropy elsewhere, typically in the non-information-bearing degrees of freedom (e.g., as heat). The erasure reduces uncertainty in the information-bearing degrees of freedom. To obey the Second Law of Thermodynamics, the system must increase entropy elsewhere. That entropy increase appears as heat dissipated into the environment—mostly affecting the non-information-bearing degrees of freedom (vibrations, kinetic energy, etc.).

**Figure 1:**
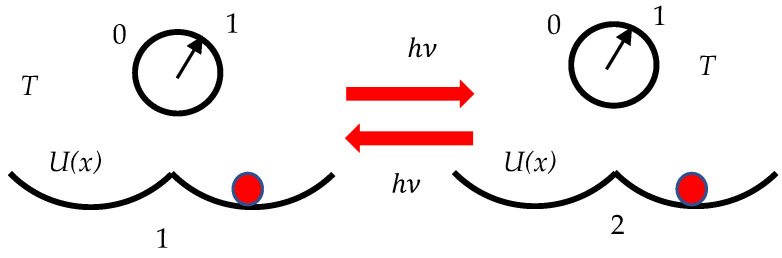
Synchronizations of clocks labeled “1” and “2” with the Einstein procedure performed with the photon hν. Both of the clocks operate under the equilibrium temperature *T.* The clocks exploit the twin-well *U*(*x*) based pendula. U(x) is the potential energy of the pendulum. Red dots indicate the location of the pendula.

The same is true for clocks. We propose to distinguish between the time-bearing and non-time-bearing degrees of freedom of the clock. The devices exploiting time-bearing degrees of freedom include timekeeping oscillators, such as pendulum or quartz crystal. The units exploiting non-time-bearing degrees are the power supply, converting mechanism and display. The minimal/elementary synchronization of the clock is equivalent to the erasure of information, necessary for zeroing the clock reading. Synchronized clocks are equivalent to the certain logical state; unsynchronized clocks are equivalent to the uncertain logical state. When we synchronize clocks (e.g., reset a pendulum location to “1” regardless of previous state of the clocks), we reduce the number of possible logical/temporal states—from two (“0” or “1”) to one (just “1”). This is illustrated with the twin-well Landauer pendulum, shown in Figure 1. Synchronization reduces uncertainty in the time-bearing degrees of freedom of the clock. After synchronization, we exactly know in what state the pendulum is located; this evidences that the clocks are synchronized. The process results in the decrease in entropy (we see a clock as the macroscopic device, operating at a certain equilibrium temperature *T)*. According to the Second Law of Thermodynamics this reduction in entropy must necessarily be compensated by an increase in entropy elsewhere, typically in the non-time-bearing *Degrees* of the clock (i.e., heat). Thus, the Landauer Principle expressed with Equation (1) becomes applicable. Let us illustrate this idea with the Einstein synchronization scheme depicted in Figure 1.

We start from a pair of clocks operating at the same temperature *T*. Consider clock “1”, exploiting the twin-well based pendulum. The pendulum may be located in the left half-well, described by the potential energy $U\left(x\right)$, corresponding to state “0”, or in the right half-well corresponding to the state “1” of the clock. The mathematical demands to the function $U\left(x\right)$ were recently treated in detail [40]. We send the photon hν towards the clock “2” exploiting the same twin-potential pendulum. The clocks may be synchronized if and only if photon hν has sufficient energy to place the pendulum in a certain (right or left half-well) regardless of the previous state of the clock “2”. Thus, Equation (2) necessarily holds:

(2) $$h\nu \geq k_{B}Tln2$$

The mass of photon, necessary for the synchronization of the clocks, is given by Equation (3):

(3) $$m_{ph}\geq \frac{k_{B}Tln2}{c^{2}}$$

It is emphasized that the introduced synchronization procedure is reversible. It does not matter what clock (“1” or “2”) emits the photon and what clock operates as an absorber of the photon. We assume that the emitting clock sends the photon ν when its state is fixed and there is no uncertainty in its ticking. If the photon is sent when the state of the emitting clock is uncertain, the total energy necessary for the synchronization of the pair is $E_{tot}=2k_{B}Tln2$. We call the entire protocol of synchronization the Einstein–Landauer procedure. This energy should be dissipated within the non-time-bearing degrees of freedom of the clock.

### 2.2. Mechanical Synchronization of the Clocks Operating at the Same Temperature

We already mentioned in the Introduction Section that mechanical antiphase synchronization was observed by Christiaan Huygens in the XVII century [3,4]. Huygens observed that two of the pendulum clocks, which were hanging from a common wooden beam placed at the top of two chairs, were showing an “odd sympathy”. Namely, the pendula of the clocks were oscillating in perfect consonance but in opposite directions, i.e., the clocks were synchronized in anti-phase. The detailed analysis of the mechanism of Huygens synchronization was reported [41,42]. It was demonstrated that, when the clocks are synchronized, the common oscillation frequency decreases, i.e., the clocks become slow and inaccurate [41]. This supports the idea that the synchronization of clocks demands energy cost and impacts their accuracy (this will be discussed below in Section 2.6).

**Figure 2:**
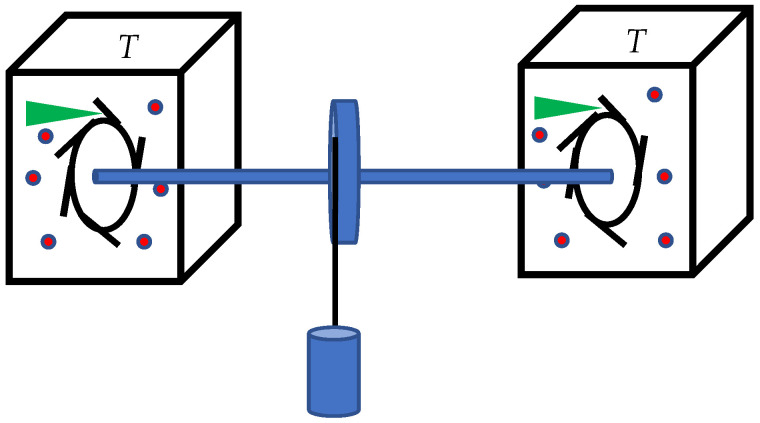
Two pairs of clocks, exploiting the folio- and verge (ratchet and pawl) escapement mechanism, depicted to operate at temperature *T*. The clocks are embedded into the ideal mono-atomic gas. Clocks are connected and synchronized with the solid elastic rod.

We focus on the thermodynamic aspects of clock synchronization. Let us illustrate the suggested thermodynamic approach with the mechanical synchronization scheme, depicted in Figure 2. Consider two pairs of clocks, exploiting folio- and verge (ratchet and pawl) escapement mechanism, depicted in Figure 2 [43]. The clocks operate at temperature *T*. For the sake of simplicity assume that the folio- and verge escapement mechanisms are embedded into the ideal, mono-atomic gas at the temperature of *T*. Operation of the folio- and verge escapement mechanism working at temperature *T* is addressed in detail in the classical textbook by Richard Feynman [44]. The clocks are synchronized by the elastic solid rod as depicted in Figure 2 (impact of the elasticity of the rod on the synchronization was investigated in detail [41,42]). The synchronization has an inherent energy cost; the minimum of this cost is defined by the thermal noise inevitable in the situation, depicted in Figure 2. The value of this cost is very roughly estimated as $E_{synchr}≅\frac{3}{2}k_{B}T,$ which is close to the Landauer limit supplied by Equation (2). This energy will be necessarily dissipated via the elastic rod, connecting folio- and verge escapement mechanisms (see Figure 2) within the non-time-bearing degrees of freedom of the clocks.

### 2.3. Energy Dissipation Within the Eddington Slow Transport of Clocks Mechanism of Synchronization

Eddington suggested the clock synchronization protocol, which is an alternative to the Einstein one [45]. This is the procedure of the slow clock-transport synchronization, which implies moving one clock slowly from one location to another [45]. Within the Eddington synchronization we (i) start with two identical clocks at the same location; (ii) synchronize clocks side by side; (iii) slowly transport one of the clocks to a distant location; and, (iv) once moved, both clocks are assumed to be synchronized. The key assumption in the Eddington synchronization is that moving the clock very slowly minimizes the time dilation effect emerging from special relativity. However, this procedure does not eliminate the stage of synchronization itself, whether optical or mechanical (such as depicted in Figure 1 or such as depicted in Figure 2). Thus, we return to the withstanding thermal noise and Landauer limit.

### 2.4. Synchronization of Clocks Operating at Different Temperatures

**Figure 3:**
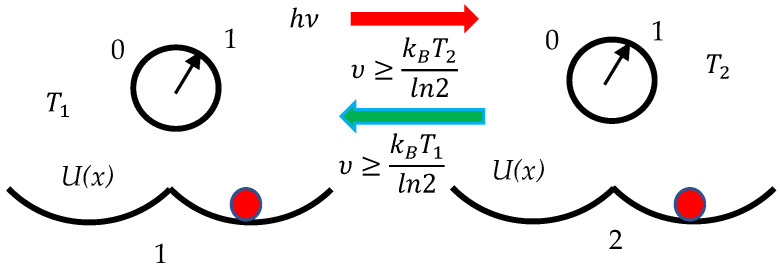
Synchronizations of clocks labeled “1” and “2” with the Einstein procedure operating at different temperatures is illustrated. The condition $T_{1}>T_{2}$ is assumed. Symmetry of synchronization is broken. Red dots indicate location of the pendula.

Consider the synchronization of clocks operating at different temperatures, illustrated with Figure 3. For the sake of unambiguity assume $T_{1}>T_{2}$.

Now the synchronization procedure turns out to be more subtle, and it does matter which clock works as an emitter of the photon and which clock absorbs the photon. When clock “1” is an emitter of the photon and there is no uncertainty in its ticking (clock “1” sends a photon when its state is fixed), Equation (4) guarantees the synchronization of the system:

(4) $$\upsilon \geq \frac{k_{B}T_{2}}{ln2}$$

When clock “2” is an emitter of the photon (we assume that there is no uncertainty in its ticking), Equation (5) provides the synchronization of the pair of clocks:

(5) $$\upsilon \geq \frac{k_{B}T_{1}}{ln2}$$

Equations (5) and (6) establish an asymmetry in the synchronization of clocks, operating at different temperatures. A very deep analogy between the transitivity of thermal equilibrium and the transitivity of clock rate synchronization was addressed in ref. [46]. We put this analogy into the context of the Landauer Principle. The Einstein–Landauer synchronization of the clocks becomes possible if Equation (6) holds, regardless which clock is emitter and which is an absorber of the photon.

(6) $$\upsilon \geq \frac{k_{B}}{ln2}max\left\{T_{1},T_{2}\right\}$$

### 2.5. Lattice of Clocks and Its Converting into Bi-Colored Graphs

**Figure 4:**
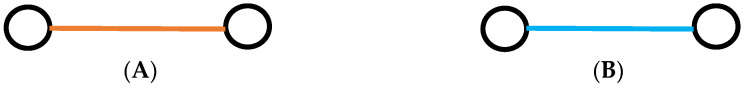
(**A**) A pair of synchronized clocks is depicted. The clocks are connected with the gold link. (**B**) A pair of non-synchronized clocks is depicted. The clocks are connected with the blue link.

Now we introduce the coloring procedure enabling converting the lattice of clocks into the bi-colored, complete graphs. Consider the two pairs of clocks depicted in Figure 4. When the clocks are synchronized with the Einstein–Landauer procedure, they are connected with the gold link (as shown in inset A); when the clocks are not synchronized, they are connected with the blue link (as shown in inset B). This coloring procedure enables representation of any lattice of clocks with the complete, bi-colored graph.

**Figure 5:**
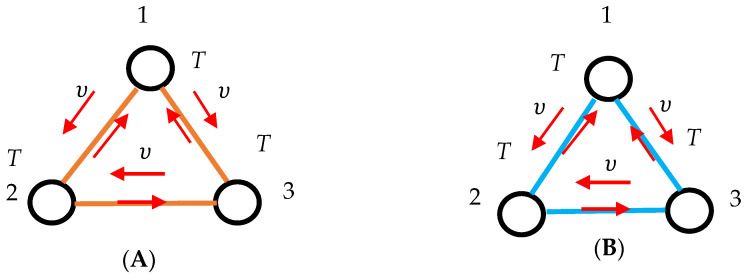
(**A**) Synchronization of a triad of clocks is depicted. The clocks operate at the same temperature *T*. Equation (3) holds. Synchronization is transitive. The entire triad is synchronized. (**B**) Synchronization of a triad of clocks is depicted. The clocks operate at the same temperature *T*. Arrows depicts photons used for synchronization. Equation (3) is not fulfilled. Non-synchronization is transitive. The entire triad is non-synchronized.

Now we put the coloring into the context of the Landauer Principle. The transitivity of synchronization becomes important, as it will be shown below. Consider two triads of clocks operating at the same temperature *T*, depicted in Figure 5.

The first triad is synchronized with the Einstein–Landauer procedure realized with photons ν. Inset “A “depicts situation, when Equation (3) is fulfilled, and photons ν enable the synchronization of the clocks. Inset “B”, in turn, illustrates the case, when $h\nu <k_{B}Tln2$ takes place and synchronization is impossible. It should be emphasized that in both of these situations (“A” and “B”) the relation “to be synchronized”/”to be not synchronized” is transitive.

It means that, if clocks “1” and “2” and “2” and “3” are synchronized with the aforementioned procedure, the clocks “1” and “3” are also necessarily synchronized. Correspondingly, if clocks “1” and “2” and “2” and “3” are non-synchronized with photon ν ($h\nu <k_{B}Tln2$), the clocks “1” and “3” are also necessarily non-synchronized with photon ν; indeed $h\nu <k_{B}Tln2$ is correct for clocks numbered “1” and “3”. Thus, any lattice of clocks, operating at the same temperature *T* and synchronized with the photons of the same frequency ν, will be necessarily completely synchronized or, alternatively, non-synchronized.

Now we address the more complicated situation. We adopt that the clocks operate at the same equilibrium temperature *T*. However, the clocks may exchange with the photons of different frequencies $\upsilon_{ik}$, where *i* and *k* are the numbers of clocks to be synchronized. Clocks numbered *i* and *k* may be synchronized when Equation (7) is true:

(7) $$h\upsilon_{ik}\geq k_{B}Tln2$$

Now the procedure of synchronization is not transitive. It means that, if clocks “i” and “k” and “k” and “l” are synchronized with the Einstein–Landauer procedure, the clocks “i” and “l” are not necessarily synchronized. Indeed, $\upsilon_{il}$ may not fulfil demands imposed by Equation (7) for the pair of clocks labeled “i” and “l”. Obviously, the relation “to be non-synchronized” is now also non-transitive. Consider a triad of clocks numbered “1” and separately “2” and “3” operating at the temperature *T*. It is possible that $h\upsilon_{12}<k_{B}Tln2$ and $h\upsilon_{23}<k_{B}Tln2$ takes place; however $h\upsilon_{13}\geq k_{B}Tln2$ holds; hence, pairs of clocks “1” and “2” and “2” and “3” are not synchronized at the time clocks “1” and “3” are synchronized. We conclude that, when the clocks may exchange with the photons of different frequencies $\upsilon_{ik}$, the relation “to be not synchronized” is not necessarily transitive.

**Figure 6:**
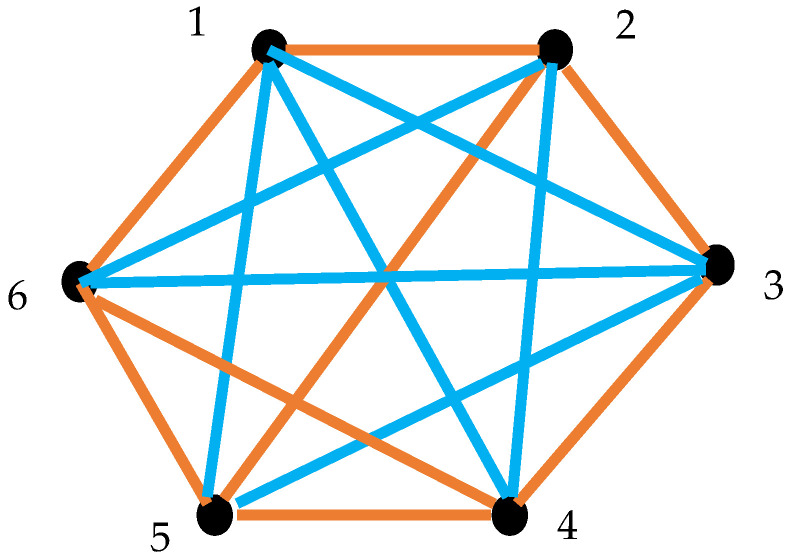
Non-transitive synchronization of the clocks with the Einstein–Landauer procedure is illustrated. The vertices of the graph numbered $\left\{1,\ldots ,\;6\right\}$ represent clocks. Synchronized clocks/vertices are connected with the gold links; non-synchronized clocks are connected with the blue links. Triangle “456” is monochromatic gold; triangle “456” represents the triad of synchronized clocks. Triangle “135” is monochromatic blue. Triangle “135” represents the triad of non-synchronized clocks.

Thus, any lattice of clocks may be described with the complete, bi-colored graph, such as that presented in Figure 6. According to the Ramsey theorem this graph should inevitably contain at least one mono-chromatic triangle. Indeed, the Ramsey number $R\left(3,3\right)=6.$ We recognize that the graph, shown in Figure 6, contains the triangle “456”, which is monochromatic gold. Hence, triangle “456” represents the triad of synchronized clocks. Triangle “135” is a monochromatic blue one. Triangle “135”, in turn, represents the triad of non-synchronized clocks.

Thus, we demonstrated the following theorem:

**Theorem** **1.** *Consider the lattice built of six clocks, synchronized with the Einstein–Landauer procedure. The clocks numbered* “i*” and “* k *” exchange with photons* $\upsilon_{ik}$*. The clocks are synchronized when* $h\upsilon_{ik}\geq k_{B}Tln2$ *holds. The clocks are non-synchronized when* $h\upsilon_{ik}<k_{B}Tln2$ *is true. The lattice inevitably contains a triad/loop of synchronized, or, alternatively, non-synchronized clocks.*

It should be emphasized that the Ramsey theory does not specify what sort of mono-colored triangle will necessarily be present in the graph. The extension of the introduced approach to the clocks operating at different temperatures is trivial, and it should be based on Equation (6).

The suggested approach is easily extended to the infinite lattice built of clocks. Consider an infinite but countable system of clocks, which form the vertices of an infinite bi-colored graph. The clocks are connected with a gold link when the clocks are synchronized, i.e., Equation (8) holds. The vertices/clocks are connected, in turn, with a blue link when the clocks are non-synchronized (demands imposed by Equation (7) are not fulfilled). According to the infinite Ramsey theorem, an infinite monochromatic (gold or blue) clique will necessarily appear in the graph [47].

We conclude that as follows:

(i) The synchronization of the lattice of clocks may be treated within the Ramsey approach.
(ii) The pair of clocks within the lattice is seen as “synchronized” or “non-synchronized”. This gives rise to the complete, bi-colored Ramsey graph.
(iii) The lattice built of six clocks necessarily inevitably contains a triad/loop of synchronized, or, alternatively, non-synchronized clocks.

### 2.6. The Landauer Limit and Accuracy of the Synchronization of the Clocks

It was demonstrated that the mechanical synchronization of clocks limits their accuracy [41,42,43]. The Landauer limit restricts the accuracy of the clock synchronization as well as the watch accuracy itself. We denote the watch accuracy of the synchronized clock ∆t. Combining the Landauer limit (see Equations (1) and (2)) with the Heisenberg uncertainty yields Equation (8):

(8) $${∆tk}_{B}Tln2≅h$$

Thus, the accuracy of the clock is given by Equation (9):

(9) $$∆t≅\frac{h}{k_{B}Tln2}$$

For the temperature of the cosmic microwave background T=2.725 K we roughly estimate $∆t≅2\times 10^{-11\;}s$ [48]. The time scale supplied by Equation (9) is close to the Planck–Boltzmann thermalization time $\tau_{PB}=\frac{h}{k_{B}T}$, which is conjectured to be the fastest relaxation timescale for thermalization of the given system [49]. The time scale Δt is much larger than the Planck time $\tau_{P}≅\sqrt{\frac{\hbar G}{c^{5}}}≅5.39\times 10^{-44\;}s$, at which quantum gravitation effects become essential, i.e., $∆t\gg \tau_{P}$ takes place, and quantum gravitation effects may be neglected under the synchronization of clocks.

## 3. Discussion

Physical clocks and computers may seem very different at first glance, but they share some deep similarities, especially in how they measure, process, and regulate information over time. Here are a few key similarities:

(i) Both are time-based systems. Computers rely on internal clocks (oscillators) to regulate operations [50,51]. Every instruction a computer executes is timed by this clock, measured in cycles per second (hertz) [50,51].
(ii) Both use regular oscillations. A quartz clock uses regular electrical oscillations to keep accurate time. A computer’s CPU has a clock signal generated by a crystal oscillator that ensures each operation is synchronized—kind of like a metronome for processing.
(iii) Deterministic behavior: Clocks and computers both operate in predictable, rule-based ways. A computer executes instructions in a fixed, logical order dictated by the program and the clock signal [50,51].
(iv) Information processing: clocks and computers both are information processing devices.
(v) Both systems move through a discrete sequence of states.
(vi) Multiple clocks (e.g., connected in a network) need synchronization for accurate timekeeping. This problem was solved by Einstein, with the procedure of synchronization exploiting the constancy of the light in vacuum. Computers also need synchronized clocks across components (CPU, RAM, and buses) to ensure proper data flow and processing.

This analogy enabled putting both computers and clocks into the paradigm of the Landauer limiting principle, which establishes the minimal cost of energy necessary for erasure of a single bit information for the computer device operating at temperature *T*. We introduced the limiting principle, which establishes the minimal energy for synchronization of clocks operating at temperature *T*. We labeled this principle as the Einstein–Landauer synchronization. The synchronization of the clocks becomes possible, when Equation (7) holds. The introduced Einstein–Landauer synchronization of clocks supports the idea that the entire universe is informational in nature, and its functioning resembles a computational process. This idea was suggested in 1989 by John Archibald Wheeler, and it was aphoristically marked as “it from bit” [52]. This very general approach to physics is intensively developed now [53,54,55].

One more important point should be addressed. It seems from the first glance that the Landauer limit may be easily broken, when a signal comes from clocks A to clock B, which is not activated yet. Thus, it is unnecessary to zero clock B under the synchronization; clock B is started by the signal coming from signal A, in which the energy is not restricted by the Landauer bound. The close inspection of the problem demonstrates that this is a wrong conclusion. Indeed, synchronization of the clocks is a loop process. The signal should be returned to clock A, which is necessarily ticking. Thus, the problem of zeroing ticking clocks remains unsolved, and the Landauer limit is unavoidable.

## 4. Conclusions

Computation, as well as measurement of time, is performed by physical devices. Rolf Landauer introduced the principle, which aphoristically is formulated as follows: “information is physical”. We suggested the principle: “time is physical”. Time is physical, because it is measured by macroscopic physical devices/clocks. These devices are necessarily discrete, and thus, resemble the computers. The clocks necessarily contain “time-bearing” and “non-time-bearing” degrees of freedom. The time-bearing degrees of freedom are pendula or electronic oscillators. The non-time-bearing degrees of freedom include energy supply, display, etc. Synchronization of clocks implies a decrease in the uncertainty in the time-bearing degrees of freedom of the clock. According to the Second Law of Thermodynamics this decrease should be compensated by the dissipation of energy in the non-time-bearing degrees of freedom of the clock. We adopt that the minimal dissipation of energy necessary for synchronization is given by the Landauer bound. It was suggested that actually the Landauer Principle represents a re-formulation of the Second Law of Thermodynamics (it is noteworthy that the demonstration of the Landauer Principle disconnected from the Second Law of Thermodynamics was demonstrated recently [40]). Consider the synchronization of the locks labeled by numbers “i” and “k”, the energy of the photon necessary for synchronization is given by the following: $h\upsilon_{ik}\geq k_{B}Tln2$, where *h* is the Planck constant. Thus, the deep relation between the Second Law of Thermodynamics and the problem of synchronization of clock is established. The synchronization of clocks needs energy; thus, the “arrow of time” emerges from the inevitable energy loss spent for the synchronization of clocks. This loss will be dissipated in the non-time-bearing degrees of freedom of the clocks. This conclusion remains untouched for the mechanical synchronization of the clocks [41,42]. We call the introduced procedure “the Einstein-Landauer synchronization” of clocks. The same results are valid for the Eddington, low-velocity transport-based synchronization of the clocks. The Einstein–Landauer synchronization restricts the accuracy of the clocks.

Lattices of clocks are addressed within the Ramsey approach. Lattices built of six clocks, synchronized with the Einstein–Landauer protocol, inevitably contain a triad/loop of synchronized, or, alternatively, non-synchronized clocks, when photons of various wavelengths are used for the synchronization. The infinite lattice built of clocks will necessarily contain an infinite clique of synchronized, or alternatively, non-synchronized clocks.

## Data Availability

The data are contained within the article.

## References

[1] Martínez A.A. (2004). Material History and Imaginary Clocks: Poincaré, Einstein, and Galison on Simultaneity. Phys. Perspect..

[2] Galison P. (2000). Einstein’s Clocks and Poincaré’s Maps: Empires of Time, Critical Inquiry.

[3] Kapitaniak M., Czolczynski K., Perlikowski P., Stefanski A., Kapitaniak T. (2012). Synchronization of clocks. Phys. Rep..

[4] Oliveira H., Melo L. (2015). Huygens synchronization of two clocks. Sci. Rep..

[5] DiSalle R. (2006). Understanding Space-Time: The Philosophical Development of Physics from Newton to Einstein.

[6] DiSalle R. (2020). Absolute space and Newton’s theory of relativity. Stud. Hist. Philos. Sci..

[7] Bussotti P., Lotti B. (2022). Newton and His System of the World. Cosmology in the Early Modern Age: A Web of Ideas. Logic, Epistemology, and the Unity of Science.

[8] Landau L., Lifshitz E.M. (1975). The Classical Theory of Fields.

[9] Tolman R.C. (1934). Relativity, Thermodynamics and Cosmology.

[10] Resnick R. (1991). Introduction to Special Relativity.

[11] Bohm D. (2025). The Special Theory of Relativity.

[12] Moller C. (1952). The Theory of Relativity.

[13] Landauer R. (1961). Dissipation and heat generation in the computing process. IBM J. Res. Dev..

[14] Landauer R. (1991). Information is physical. Phys. Today.

[15] Landauer R. (1996). Minimal energy requirements in communication. Science.

[16] Bennett C.H., Landauer R. (1985). The fundamental physical limits of computation. Sci. Am..

[17] Maroney O.J.E. (2005). The (absence of a) relationship between thermodynamic and logical reversibility. Stud. Hist. Philos. Sci. B.

[18] Piechocinska B. (2000). Information erasure. Phys. Rev. A.

[19] Parrondo J.M.R., Horowitz J.M., Sagawa T. (2015). Thermodynamics of information. Nature Phys..

[20] Sagawa T. (2014). Thermodynamic and logical reversibilities revisited. J. Stat. Mech..

[21] Herrera L. (2014). The mass of a bit of information and the Brillouin’s principle. Fluct. Noise Lett..

[22] Herrera L. (2020). Landauer Principle and General Relativity. Entropy.

[23] Bormashenko E. (2019). Generalization of the Landauer Principle for Computing Devices Based on Many-Valued Logic. Entropy.

[24] Vopson M. (2019). The mass-energy-information equivalence principle. AIP Adv..

[25] Müller J.G. (2024). Events as Elements of Physical Observation: Experimental Evidence. Entropy.

[26] Bormashenko (2019). The Landauer Principle: Re-Formulation of the Second Thermodynamics Law or a Step to Great Unification?. Entropy.

[27] Maroney O.J.E. (2009). Generalizing Landauer’s principle. Phys. Rev. E.

[28] Esposito M., Van den Broeck C. (2011). Second law and Landauer principle far from equilibrium. Europhys. Lett..

[29] Bérut A., Arakelyan A., Petrosyan A., Ciliberto S., Dillenschneider R., Lutz E. (2012). Experimental verification of Landauer’s principle linking information and thermodynamics. Nature.

[30] Lairez D. (2023). Thermodynamical versus Logical Irreversibility: A Concrete Objection to Landauer’s Principle. Entropy.

[31] Buffoni L., Campisi M. (2022). Spontaneous Fluctuation-Symmetry Breaking and the Landauer Principle. J. Stat. Phys..

[32] Bondy J.A., Murty U.S. (2008). R Graph Theory.

[33] Chartrand G., Chatterjee P., Zhang P. (2023). Ramsey chains in graphs. Electron. J. Math..

[34] Chartrand G., Zhang P. (2021). New directions in Ramsey theory. Discrete Math. Lett..

[35] Graham R.L., Rothschild B.L., Spencer J.H. (1990). Ramsey Theory.

[36] Graham R., Butler S. (2015). Rudiments of Ramsey Theory.

[37] Di Nasso M., Goldbring I., Lupini M. (2019). Nonstandard Methods in Combinatorial Number Theory, Lecture Notes in Mathematics.

[38] Katz M., Reimann J. (2018). Introduction to Ramsey Theory: Fast Functions, Infinity, and Metamathematics.

[39] Bormashenko E.d., Shvalb N. (2024). A Ramsey-Theory-Based Approach to the Dynamics of Systems of Material Points. Dynamics.

[40] Witkowski K., Brown S., Truong K. (2024). On the Precise Link between Energy and Information. Entropy.

[41] Peña Ramirez J., Olvera L., Nijmeijer H., Alvarez J. (2016). The sympathy of two pendulum clocks: Beyond Huygens’ observations. Sci. Rep..

[42] Peña Ramire J., Aihara R., Fey R.H.B., Nijmeijer H. (2014). Further understanding of Huygens’ coupled clocks: The effect of stiffness. Phys. D.

[43] Blumenthal A.S., Nosonovsky M. (2020). Friction and Dynamics of Verge and Foliot: How the Invention of the Pendulum Made Clocks Much More Accurate. Appl. Mech..

[44] Feynman R. (1964). The Feynman Lectures on Physics.

[45] Bormashenko E. (2025). Physical and Logical Synchronization of Clocks: The Ramsey Approach. Foundations.

[46] Zheng Z., Chen P. (1997). Zeroth law of thermodynamics and transitivity of simultaneity. Int. J. Theor. Phys..

[47] Li Y., Lin Q. (2020). Elementary Methods of the Graph Theory.

[48] Fixsen D.J. (2009). The Temperature of the cosmic microwave background. Astrophys. J..

[49] Hartnoll S.A., Mackenzie A.P. (2022). Colloquium: Planckian dissipation in metals. Rev. Mod. Phys..

[50] Lamport L. (1978). Time, clocks, and the ordering of events in a distributed system. Commun. ACM.

[51] Kulkarni S.S., Demirbas M., Madappa D., Avva B., Leone M., Aguilera M.K., Querzoni L., Shapiro M. (2014). Logical Physical Clocks. Principles of Distributed Systems, Proceedings of the 18th International Conference, OPODIS 2014, Cortina d’Ampezzo, Italy, 16–19 December 2014.

[52] Wheeler J.A. Information, physics, quantum: The search for links. Proceedings of the 3rd International Symposium on Foundations of Quantum Mechanics in the Light of New Technology.

[53] Vopson M. (2025). Is gravity evidence of a computational universe?. AIP Adv..

[54] Vopson M. (2020). The information catastrophe. AIP Adv..

[55] Bormashenko E. (2020). Informational Reinterpretation of the Mechanics Notions and Laws. Entropy.

